# Comprehensive analysis of Australia’s aged care system to inform policies for a sustainable future

**DOI:** 10.3389/fpubh.2025.1525988

**Published:** 2025-04-11

**Authors:** Turki Alsaeed, Tracy Washington, Bo Xia

**Affiliations:** School of Agriculture and Built Environment, Faculty of Engineering, Queensland University of Technology, Brisbane, QLD, Australia

**Keywords:** Australian aged care, population ageing, ageing policies, aged care sector, aged care reforms

## Abstract

**Background:**

Australia’s aging population presents significant socioeconomic challenges, necessitating the aged care sector reforms. Projections indicate continued growth in this demographic, prompting the implementation of various funding mechanisms to support healthy aging. However, notable disparities persist, including care quality concerns, workforce shortages, and safety issues, hindering the sector’s ability to meet consumer expectations. Despite recognition of these challenges, no comprehensive overview exists addressing these shortcomings. This study aims to provide a comprehensive analysis of the literature to identify these challenges and inform policy development.

**Methods:**

In this study, a scoping review was conducted, examining primary and secondary sources, including peer-reviewed articles, government reports, and aged care policies. The Scopus database was searched using relevant keywords, and a snowball search technique was employed to identify additional literature. The inclusion criteria were applied, and journal articles were screened for titles and abstracts before full-text analysis. Thematic analysis was conducted on selected literature, and secondary data were from administrative and organizational websites and agencies.

**Results:**

Findings indicate a preference for home-based care among older Australians due to concerns about quality and safety in aged care facilities. While the Australian government has developed reforms and policies to govern the sector, funding remains insufficient to meet the escalating demand for high-quality care. Challenges include workforce shortages, the growing burden of aging, and difficulties in adopting emerging technologies, which impact the quality of care delivered to older Australians.

**Conclusion:**

This paper serves as a resource for policymakers and aged care professionals, informing the development of reforms to address pressing issues in the sector. A comprehensive evaluation of existing knowledge provides a clearer understanding of current and future obstacles ensuring a holistic view and fostering the development of sustainable aged care reforms.

## 1 Introduction

Population aging represents a major socioeconomic transformation that profoundly affects nearly all nations globally. Presently, individuals aged 65 years and above constitute 10% of the global population and estimates suggest this figure will rise to 16% in the coming years (1). Projections indicate that the global demographic of people aged 65 years and above will virtually double, while those aged 80 years and older will experience a threefold increase. In Australia, the proportion of individuals aged 65 and above already stands at 16.7% and is projected to rise from 4.31 million in 2021 to 6.66 in 2041 (1). To effectively address this demographic shift and the multifaceted health conditions and disabilities associated with aging, the Australian aged care system receives substantial investment, with the Commonwealth government allocating AUD 24.8 billion in 2021–22 alone (2) to promote healthy aging and enhance the quality of life for older adults (3). In particular, among the Organization for Economic Co-operation and Development (OECD) countries, Australia has the highest proportion of individuals receiving aged care (4). Currently, an estimated 1.3 million Australians access aged care services, and 5% of the aged population in Australia resides in institutionalized aged care facilities (5). Most older adults prefer to live in their own homes or familiar communities rather than in institutions, benefiting from greater independence and well-being (6). As a cost-effective alternative, governments actively promote aging-in-place over institutionalized care.

Subject to stringent regulations, aged care services in Australia—mainly including residential, in-home, and short-term care, have witnessed substantial reforms over the past decade (3). The government subsidizes most aged care services (7), rendering them accessible to non-indigenous individuals aged 65 and above and Indigenous people aged 50 and above. The aged care sector offers services to its residents by ensuring their safety and well-being, providing necessary support for families, and alleviating pressure on healthcare systems (8). It helps older adults maintain independence by assisting with daily activities, allowing them to stay in their homes longer and preserve autonomy (9). In addition to physical health, aged care services address emotional and social well-being by reducing loneliness through social programs and companionship. By addressing both health and social needs, these services enhance the quality of life for older adult individuals and contribute to healthier, more resilient communities (8, 9).

Along with the increasing importance of the aged care system to Australian society, the Australian Aged Care system is grappling with significant challenges, with persistent issues such as inadequate services, poor quality, accountability deficits, and gaps in monitoring and reporting continue to raise concerns (2). Disparities in aged care access persist between urban and non-urban populations, with regional, remote, and rural areas often facing limited access to aged care services, which have been associated with a higher prevalence of preventable diseases among older adults (7). Furthermore, the prevailing level of care has failed to meet societal expectations although expectations for improved outcomes remain high. Hibbert et al. linked the shortcomings of the aged care sector to its overwhelming financial burden, weak regulations, and the exclusion of consumer voices in service design and delivery, all worsened by widespread societal ageism (10).

Addressing these challenges necessitates fundamental reforms to the Australian Aged Care system. However, it will not be achieved without a comprehensive overview of the current aged care system, which is missing from the current body of knowledge. In response, the present study aims to undertake a comprehensive examination of various perspectives and insights gleaned from the peer-reviewed literature, government policies, industry reports, and Australian aged care websites. By fostering a more nuanced understanding of the ramifications of population aging and the aged care system in Australia, this research seeks to inform policy implications that can strengthen both the current and future aged care systems.

## 2 Methods

This study employs a content analysis methodology (11) to examine existing peer-reviewed literature and administrative databases concerning Australia’s aged care system. This approach enables an evaluation of the existence and depth of available research on the topic while facilitating the inclusion of diverse research designs and data sources to better understand the structure of the aged care system.

The study draws upon information gathered through database searches in Scopus, as well as manual searches of relevant websites. The keywords utilized for the literature search were “aged care,” “aged care services,” “aged care system,” “aged home care,” “Australia,” “people-centred care,” “care structure,” “care delivery.” In addition, due to the inherent differences between databases, a snowball searching technique was applied, which involved reviewing the reference lists of relevant articles and leveraging databases and search engines to identify related articles (12). This method allowed for overcoming variations in terminology and focusing on the function, purpose, structure, and operation of the aged care system by using combinations and modifications of keywords in free-text searches. Moreover, this approach enhanced the yield of articles pertinent to the study’s objectives.

Academic literature was considered eligible for inclusion if it pertained to the aged population and aged care system and was published in English between January 2015 and August 2024 (date of the search) to maintain relevance to the contemporary context of aged care services. The choice of this timeframe ensured that the findings were pertinent to the current aged care service and policy landscape. Studies concerned with associated systems offering palliative, end-of-life, and hospice services were excluded due to their distinct user bases and objectives. Furthermore, studies focused on highly specific contexts or populations, such as services tailored to veterans, were deemed less relevant to the broader aged care system in Australia and were thus excluded. Lastly, dissertations were excluded unless their findings had been published.

### 2.1 Screening, selection, and analysis

Titles and abstracts of journal articles were screened based on the predefined search and exclusion criteria. Articles deemed relevant after this initial screening underwent a comprehensive full-text assessment, yielding a substantial body of pertinent literature. The inclusion criteria were subsequently reevaluated and refined after screening titles and abstracts to optimize the selection process. The included studies demonstrated a clear focus and relevance, contributing valuable findings. A thematic analysis was then conducted on the selected studies. Initially, all studies were closely read to gain an understanding of the data. Key findings from each study were systematically identified and coded. These codes were carefully analyzed and organized into themes, which informed the review’s development. This rigorous analytical process enabled the synthesis of diverse perspectives and facilitated an in-depth exploration of Australia’s aged care system.

In addition to scholarly articles, this review incorporated secondary data analysis, which is defined as the examination and analysis of the data, either previously published or the original data, by researchers who did not participate in the original data collection process (13). Data was sourced from various administrative and organizational entities involved in Australia’s aged care system using the same search words on their websites. Relevant agencies and organizations of interest included the Australian Institute of Health and Welfare, the Australian Government, the Australian Parliament, the Aged Care Financing Authority, the Aged Care Quality and Safety Commission, the Senior Rights Service, National Aged Care Alliance, Law Council of Australia, Departments of Health, Department of Health and Aged Care, and respective acts and regulations. The integration of these sources allowed for the exploration of new data patterns and the development of a comprehensive understanding of the structure and operation of the aged care system in Australia. These secondary data sources provided valuable statistical information regarding the financial structure of aged care and the demographics of older adults receiving different types of aged care services. Secondary data sources provided a valuable opportunity to access extensive and reliable databases as primary data collection in the aged care system can be resource intensive; secondary data offers a more practical and efficient solution. This multi-faceted approach to data collection and analysis facilitates a comprehensive examination of Australia’s aged care system.

## 3 Results

### 3.1 What we know about the aged care system from the literature review

#### 3.1.1 Evolution of the aged care concept

Aged care and old age have been explored in general for centuries. The realization that aged people needed closer attention and care led to an increasing focus on aged care in the 20th Century. In the West, medical and social care for older adults started gaining attention in the 19th Century, steered by the increasing numbers of the aged. In the beginning, women and churches started operating alms-houses, where the old could go to be taken care of, but in which they lived alongside other less fortunate members of society, such as the homeless and the mentally ill (14). By the 20th Century, Denham points out that aged care had started from the intervention of churches in the West, which felt guilty that the older people spent most of their last days around the mentally ill, criminals, and “unsavory” individuals. Initially, only seniors recognized as members of a particular faith were accepted into the aged care facilities run by churches. The United States (U.S.) government recognized the need for further interventions in aged care in the 1920s, with organizations opening care facilities under different conditions and coordinating with the government (14). With the government’s intervention, more facilities were opened to care for the aged, and more stakeholders took part in the aged care process. Following government engagement, the aged care sector grew significantly between the 1950s and the 1980s. In the U.S., legislation such as the Medical Facilities Survey and Construction Act of 1954 influenced the development of public institutions that would care for the neediest cases among older adults (14). Likewise, other countries, such as the United Kingdom, Australia, and Germany, also experienced increasing popularity of aged care systems over the latter part of the 20th Century (15). However, industry investigations conducted in the 1970s showed that most of the facilities offered substandard care, mostly lacking instrumental elements such as medical care, food, and enough attendants, a situation that made them be considered as halfway homes between the living and the cemetery (15). In the late 1970s, the government increasingly engaged in policy development to control the quality of care delivered in homes for the aged, leading to increased demand for aged care facilities. Over the subsequent years, the popularity and quality of aged care facilities across the globe have grown, with more people opting to spend their advanced years in such homes (15).

#### 3.1.2 Historical mapping of the Australian aged care system: how did it emerge?

The history of aged care in Australia can be traced to the creation of the Benevolent Society of New South Wales in 1815. The group started establishing large indoor-relief institutions. However, these efforts lacked legal support (16). The attention given to aged care remained subdued. The key element identifying the aged people was their poverty because they could not participate as members of the workforce in a capitalist environment (17, 18). After all, few reached retirement age in the early 19th century, and most of the older adults were cared for by family members (19). Government social planning remained haphazard and piecemeal. From the 1950s, aged care facilities consisted of nursing homes and hostels for seniors, served by registered nurses (RNs) and nursing aides. These were run by state governments, charities, and church-based institutions, and some were businesses, but it was the homes that churches provided to their members that formed the prototypes of later aged-care systems (20). The federal government started with the Aged Persons Homes Act (21), which provided grants to the voluntary sector mainly in the lead-up to the election (21, 22). However, population aging was not a major policy concern amid the massive baby boom and migration at a time when the term ‘old age’ was perceived as synonymous with ‘pensioners’ (22, 23). The adequacy of aged care continued to vary greatly even after nursing homes were made eligible for Commonwealth government subsidy in 1962 (24, 25). It was not until 1982, when the Aged Care Coalition was formed, that older residents’ rights to aged care gained prominence in national policy debates (26). The rapid aging of the post-war baby boom generation has increasingly strained social security systems as they reach older ages (27–29).

Increased costs of aged care in the 1990s prompted the need to review regulations, funding, and staffing costs. In a bid to encourage investments in aged care services, the 1997 Aged Care Act required the government to fund aged care expenditures and placed a cap on aged care funding (30). Despite these advances, concerns over standards, dysfunction, abuse, and neglect remained. Multiple inquiries and hearings on aged care quality and safety have been undertaken, and major reforms have been recommended (31–33). Despite the advances in aged care, the need to reform the aged care system remains to address persistent accountability, governance, and funding concerns.

#### 3.1.3 Australian aged care sector landscape

##### 3.1.3.1 Aged care categories

The Australian government classifies aged care into three categories: residential, in-home, and short-term care (34). The classification is shared by states and determines how providers deliver aged care services (35–37). Residential care provides general care and support in aged care homes (38). This type of care is suitable when one cannot live independently at home (39). Also referred to as assisted living or personal care, residential care homes host multiple older people collectively and offer services such as dressing, washing, administering, and giving medication (40). As Seah et al. and Gilbert et al. observed, the extent of service should depend on the personal needs of patients (41, 42). Even though Dyer et al. raised concerns about the reliance on residential aged care services (43), Kalaitzidis and Harrington, as well as Minney and Ranzijn, acknowledged positive feedback from aged care residents’ perspectives (44, 45). In-home care involves delivering care in the patient’s own home. This type of care applies when a resident needs extra help even though they are still living independently (46, 47). This option is convenient when family members are not accessible. Ding et al. and Visvanathan et al. observed that seniors prefer to remain at home if essential specialist care services and support are available (48, 49). Short-term care is synonymous with flexible care that depends on an individual’s needs. This type of care is common following a hospital stay or a period of illness. It is also an option when families lack the resources to offer aged care for a period of time. The purpose of short-term care is to help seniors cope with life interruptions (50, 51). There are multiple and varied ways of delivering short-term care, depending on one’s situation and needs. Among the examples are short-term restorative care designs to assist in slowing difficulties faced in everyday tasks or returning to past levels of independence (50–53). Another way includes transition care that aims to assist seniors’ recovery after hospital stays (50). Care delivery stops once the patient regains functional independence and confidence. Short-term care can also be in the form of respite care, which enables family members or caregivers to take a break for a set period (54, 55). It involves a short-term agreement in which a professional caregiver delivers care to a patient in their own home (56, 57).

The range of care types offered in the Australian Aged care sector involves various services. In home-based care, the offered services aim to enable seniors to be more independent at home. For instance, the government runs the Commonwealth Home Support Programme (CHSP), in which the government pays care providers to offer transport, shopping, and cleaning services to seniors (58). The government also finances home care packages that bundle personal care with medication management, nursing, social support, and domestic assistance, depending on individuals’ needs. In residential aged care, several unique services are offered. The sector offers accommodation services, catering, and laundry services (59). These accompany the personal care, nursing care, and health services available throughout. Most residential homes also have facilities that enable seniors to engage in recreational and social activities (5). Even in short-term care situations, respite care, palliative, and transition care are characteristic services in the sector (60). The sector also has specialists to offer dementia care services to seniors (61, 62). The sector is also involved in addressing the specific needs of aged veterans (63). The wide range of services by the Australian aged care sector helps seniors maintain their independence, where possible, and enhances their quality of life and well-being.

##### 3.1.3.2 Eligibility overview

Eligibility for subsidized aged care depends on mandated assessment results. A senior must meet the set eligibility criteria to access aged care services. One must be aged 65 years or older to qualify for the Aged Care Assessment (64). Studies also utilize this age criterion as the primary criterion in assessing eligibility (60, 65–67). Notably, eligibility criteria can extend even below 65 years (40, 68, 69). Aboriginal or Torres Strait Islander persons are eligible if 50 years of age or older (70). However, the age requirement is not sufficient to secure access to aged care services from the government. A needs assessment is necessary to determine the extent of assistance that one needs. The needs assessment determines a senior’s capacity to handle daily tasks independently (71, 72). The Australian Government and MyAgedcare show that exceptions also apply for low-income and homeless people at risk of homelessness once they are 50 years old or older, which Walsh et al. supported because homelessness afflicts physical and mental conditions and functional impairments (73, 74). This exception also applies to the vulnerable members of minority groups aged 45 years or older (75). Assessment for eligibility begins with age, but a needs assessment contributes significantly to determining the specific aged care services that an individual qualifies for.

#### 3.1.4 Current development of the Australian aged care industry

##### 3.1.4.1 A comprehensive overview of Australian aged care industry regulations

The aged care system is subject to several policies. The privacy policy outlining the practices of handling personal information would apply as it applies to any other health and social care. The Privacy Act 1988 and Australian Privacy Principles dictate personal information handling (76). The growth of the aging population is revolutionizing the aged care system. Privacy policy is highly relevant to the aged care system because older individuals often require extensive health and social care services, which involve the collection, use, and storage of sensitive personal information (77). This information may include their place of residence, household members, health conditions, financial situation, and vulnerabilities. The Department of Health and Aged Care emphasizes that aged care organizations must protect the private and sensitive information of individuals receiving care (78).

Maintaining accurate and up-to-date records is essential for aged care organizations to deliver appropriate services, provide tailored support, and ensure correct billing. As aged care staff interact with residents, they often gather additional personal details, which are documented to enhance the quality of personalized, person-centred care. However, it is the organization’s responsibility to ensure that all personal and sensitive information is securely managed and protected. To safeguard the rights of consumers, the Australian Government has implemented the Privacy Act 1988, which contains 13 Australian Privacy Principles (APPs) that set clear guidelines on how aged care providers must manage personal information (77, 79). Aged care facilities and service providers are required to obtain informed consent before collecting or sharing personal data, ensure secure storage of records, and allow individuals to access and correct their information when necessary. The Government enforces privacy protections through regulatory bodies such as the Office of the Australian Information Commissioner (OAIC) (80), which investigates complaints and ensures compliance. Additionally, sector-specific frameworks like the Aged Care Quality Standards reinforce privacy protections as a fundamental aspect of quality care as outlined in the Aged Care Quality and Safety Commission Act 2018 and the Privacy Act 1988 (77, 79). The current policy environment reflects the increasing demands attributable to the rapid growth of the aging population in recent decades (81). As the country witnessed the phenomenon in the 1990s, the parliament enacted the Aged Care Act in 1997, the foundational legislative framework (82). It forms the basis of regulations, quality standards, administration, and funding of the aged care sector. The Act designated the Commonwealth government as responsible for aged care policy, regulation, and funding (83). The Act reframed the role of residential aged care services and included provisions underpinning the expansion of aged care services to enable the aged to remain in their homes.

Attempts to reconceptualize residential aged care unintentionally make it challenging to enforce quality and health as desired. The expansion of aged care services raised the level and complexity of needs that one must meet before receiving residential aged care (49, 84). The Aged Care Act 1997 made the aged care system in Australia highly regulated at the top. The Federal government funds the Aged Care Assessment Programme to identify individuals’ needs and determine eligibility for and access to commonwealth support services. An Aged Care Assessment Team (ACAT), including clinicians, assumes the responsibility of conducting the assessment. Approved service providers must be accredited after adherence to existing Aged Care Quality Standards (85). Gaps arise because a single centralized authority, the Australian Department of Health and Aged Care, assumes the quality monitoring and inspection mandate, but the management and organizational structure of individual ACATs are not uniform. They differ across districts because of conflicting governance, funding, internal processes, and organizational structures (3). In addressing identified gaps, the Aged Care (Living Longer Living Better) Act 2013 recommended supporting seniors to stay at home, improving the quality and value of services, enhancing services to the vulnerable, and improving information access (86). The goal was to increase consumers’ choices and enhance market efficiency and competitiveness. The Aged Care Legislation Amendment (Increasing Choice in Home Care) Act 2016 followed and came into effect in 2017 to increase market-based decision, choice, and competition. It redirected funding to consumers to ease the change of providers and improve the prioritization of services (87, 88).

In 2014, new supporting principles were introduced to guide care providers and are relevant to the Aged Care Sector. The Accountability Principles 2014 were established to guide the preparation and approval of financial reports and ensure personnel suitability (89). Quality of Care Principles 2014 were developed to ensure the maintenance of quality care and guide provider accreditation (90). These supporting principles were accompanied by Information Principles, Sanction Principles, Records Principles, and Information Principles (91). The Aged Care Quality and Safety Commission works under the Aged Care Quality and Safety Commission Rules 2018 and the Aged Care Quality and Safety Commission Act 2018. Together with the Aged Care Act 1997, the Commission Act and Rules inform the compliance and enforcement policy that defines how the Commission should apply its powers and approach to compliance and enforcement to address sector-wide risks and shape market behavior (92).

Alongside such policy gaps, the aged care system has been marred with allegations. Cases of neglect, abuse, poor service, and sub-standard service delivery are common (7). The average quality of personal care workers has been declining. Similarly, the incentive to invest in systems that can capture, and monitor needs and outcomes has declined (85). As highlighted in the Royal Commission into Aged Care Quality and Safety (2018–2021), policy gaps in the Australian aged care sector, primarily due to issues including inadequate and untrained workforce, and insufficient funding have led to instances of neglect, abuse, poor service, and substandard care delivery, indicating systemic failures in regulatory enforcement. Cases of residents experiencing verbal or psychological abuse have been well-documented (93), Furthermore, weak enforcement and inconsistent oversight of the Aged Care Quality Standards have allowed some providers to continue operating despite repeated violations.

The Aged Care Act 1997 (94) serves as the primary legislative framework governing aged care in Australia. It establishes the rights of residents and the responsibilities of providers. Other key regulations include the Aged Care Quality Standards (95), which guarantees residents dignity, privacy, and quality service, and the Charter of Aged Care Rights (96), which outlines expectations for care. Cases of elder abuse can also be prosecuted under the Crimes law and relevant state and territory laws, as outlined by the Australian Law Reform Commission (97). When disputes arise, several mechanisms exist to address complaints. The Aged Care Quality and Safety Commission investigates complaints from residents, families, staff, and healthcare providers, and takes appropriate steps to resolve them through discussion, mediation, and conciliation (98). Additionally, the national ELDER Help free helpline (1800), and legal state and territory help are available for individuals who provide information about abuse and abuse prevention. In cases when issues are not resolved, Commonwealth Ombudsman services are further available (99).

**Figure 1:**
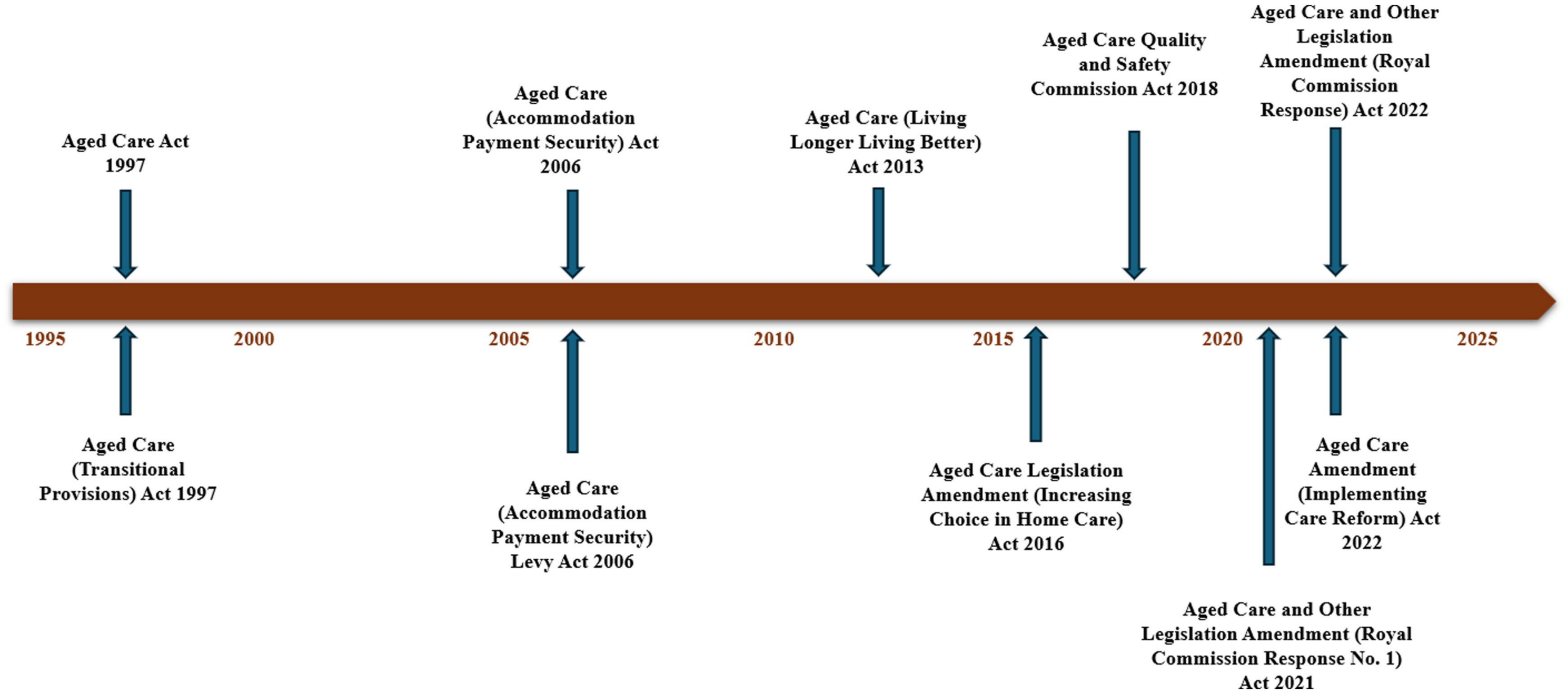
Australian aged care sector legislation timeline. Source: Aged Care (Living Longer Living Better) Act 2013 (86), Aged Care Legislation Amendment (Increasing Choice in Home Care) Act 2016 (87), and Australian Government (157, 158).

In response to the Royal Commission’s Final Report, the Australian Government has introduced significant reforms aimed at addressing policy gaps and improving the aged care sector. These reforms include measures to tackle workforce shortages and enhance service delivery (100). While these reforms represent a positive step forward, sustained monitoring, rigorous enforcement, and continuous improvement remain critical to ensure that aged care residents consistently receive the high-quality, dignified care they deserve. The Australian government mandated the Royal Commission into Aged Care Quality and Safety to enhance the system’s sustainability and ensure appropriate care for older adults. In its report, the Royal Commission unearthed the shortcomings in the system and made recommendations to fundamentally reform it (2). Several attempts at policy reforms on providing aged care services have been made as demands for aged care increase (101, 102). Figure 1 indicates several aged care laws governing the Australian aged care sector.

#### 3.1.5 Residents profile

**Figure 2:**
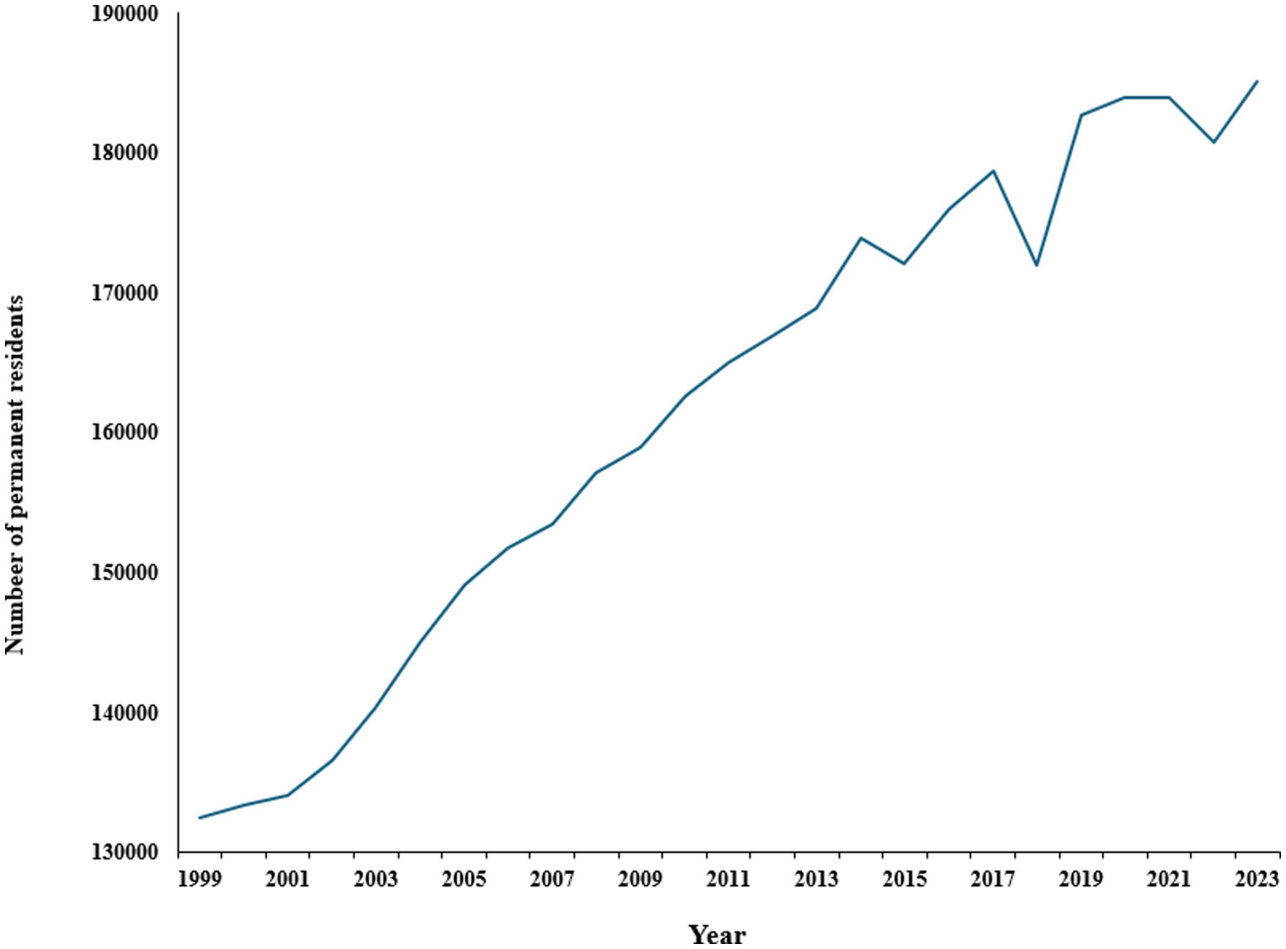
Number of permanent residents living in Australian aged care facilities. Source: Australian Institute of Health and Welfare (103, 104).

The census data on the number of permanent residents living in Australian aged care facilities from 1999 to 2023 shows a general upward trend, reflecting the increasing demand for aged care services over this period (103). As shown in Figure 2, in 1999, there were 132,420 residents, and by 2023, this number had risen to 185,127, representing a significant overall increase of 39.9%. The number of residents grew consistently with notable peaks between 2003 and 2004, showing an increase of 4,697 residents, while from 2018 to 2019, there was a substantial rise of 10,705 residents. However, the data also indicates some fluctuations with a slight decrease in the number of residents in 2015 and again in 2018. More recently, after reaching 183,894 in 2021, the number dipped to 180,750 in 2022 before rising again to 185,127 in 2023. These fluctuations can be attributed to various factors, including uneven older residents’ distribution across Australia, the impact of the COVID-19 pandemic, changes in healthcare policies, and broader demographic trends (104, 105).

**Figure 3:**
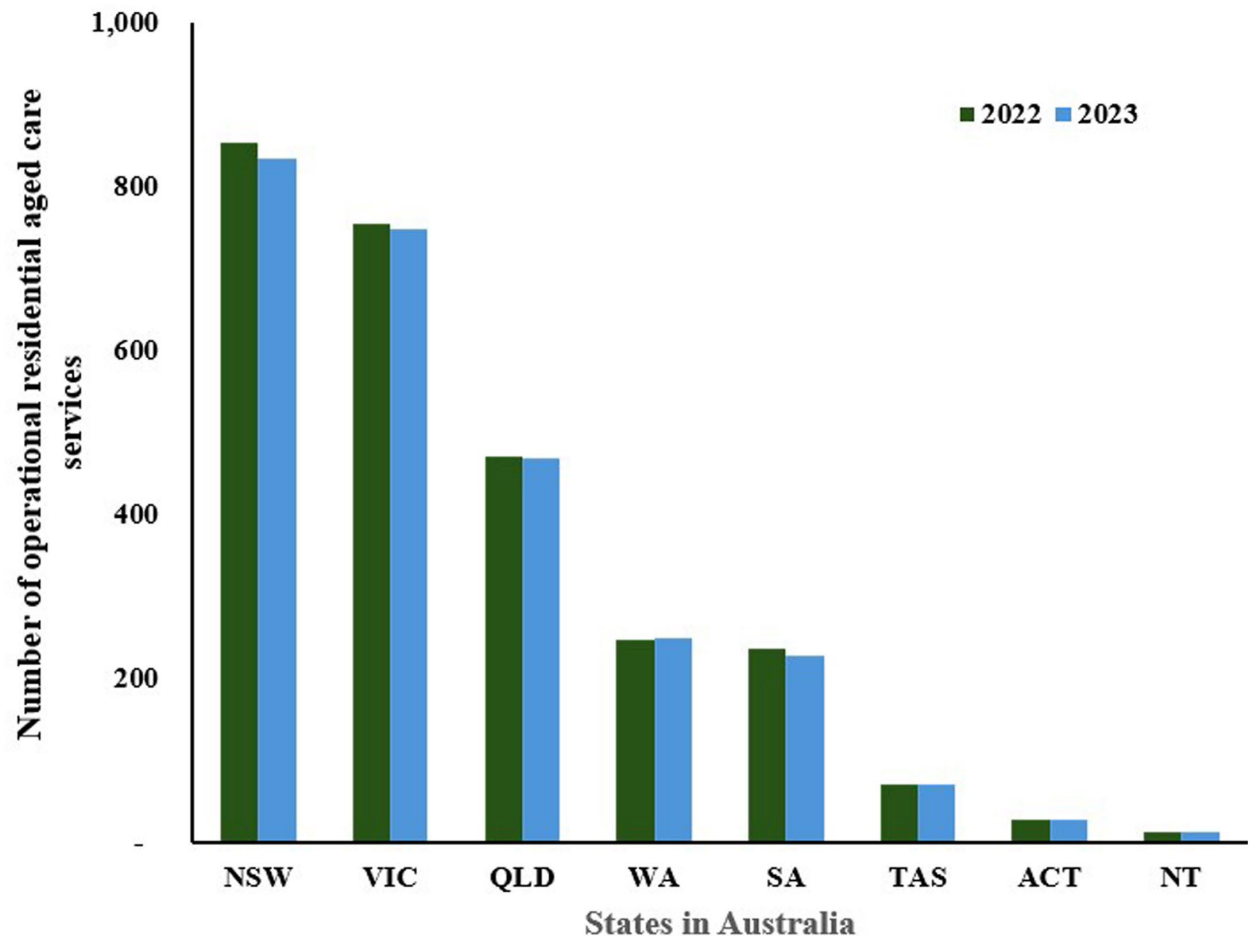
Number of residential aged care services in Australia, by state 2022–2023 (NSW: New South Wales, VIC: Victoria, QLD: Queensland, WA: Western Australia, SA: South Australia, TAS: Tasmania, ACT: Australian Capital Territory, NT: Northern Territory). Source: Australian Institute of Health and Welfare (103).

**Figure 4:**
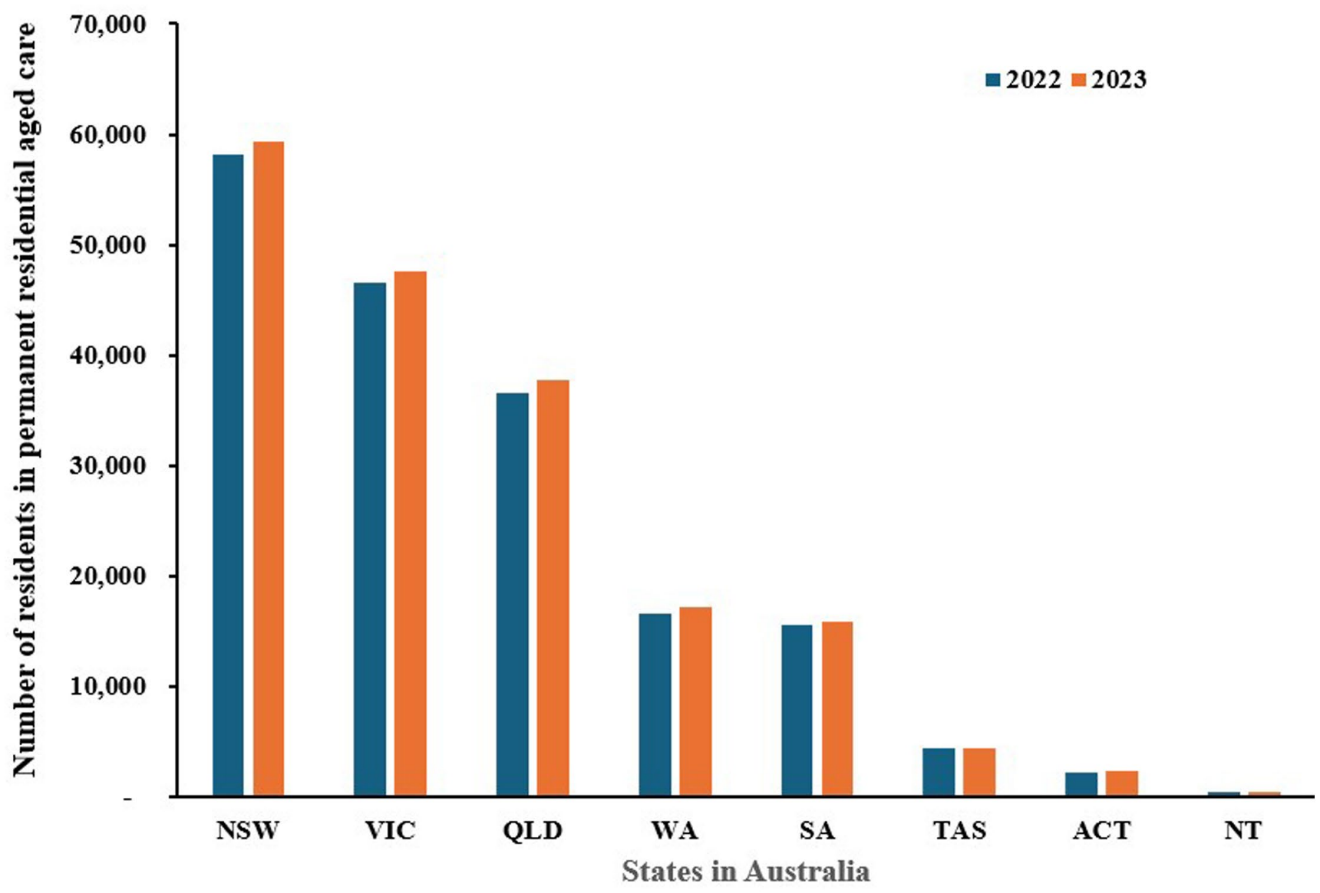
The number of people living in permanent residential care by state in Australia, 2022–2023 (NSW: New South Wales, VIC: Victoria, QLD: Queensland, WA: Western Australia, SA: South Australia, TAS: Tasmania, ACT: Australian Capital Territory, NT: Northern Territory). Source: Department of Health and Aged Care (106, 107) and Australian Institute of Health and Welfare (159).

Figure 3 shows the number of operational residential aged care services across different states and territories for the years 2022 and 2023 while Figure 4 indicates the number of residents living in these permanent residential cares (106, 107). Overall, the data indicates a slight decrease in the number of residential aged care services in most states, suggesting a trend away from institutional care. For example, New South Wales (NSW) saw a decrease from 853 facilities in 2022 to 835 in 2023, while Victoria (VIC) dropped from 754 to 748 in the same period. Similarly, South Australia (SA) reduced its number from 237 to 229. Conversely, there was a slight increase in Western Australia (WA), from 247 to 249, while other smaller states like Tasmania (TAS) and territories such as the Australian Capital Territory (ACT) and Northern Territory (NT) maintained stable numbers.

**Figure 5:**
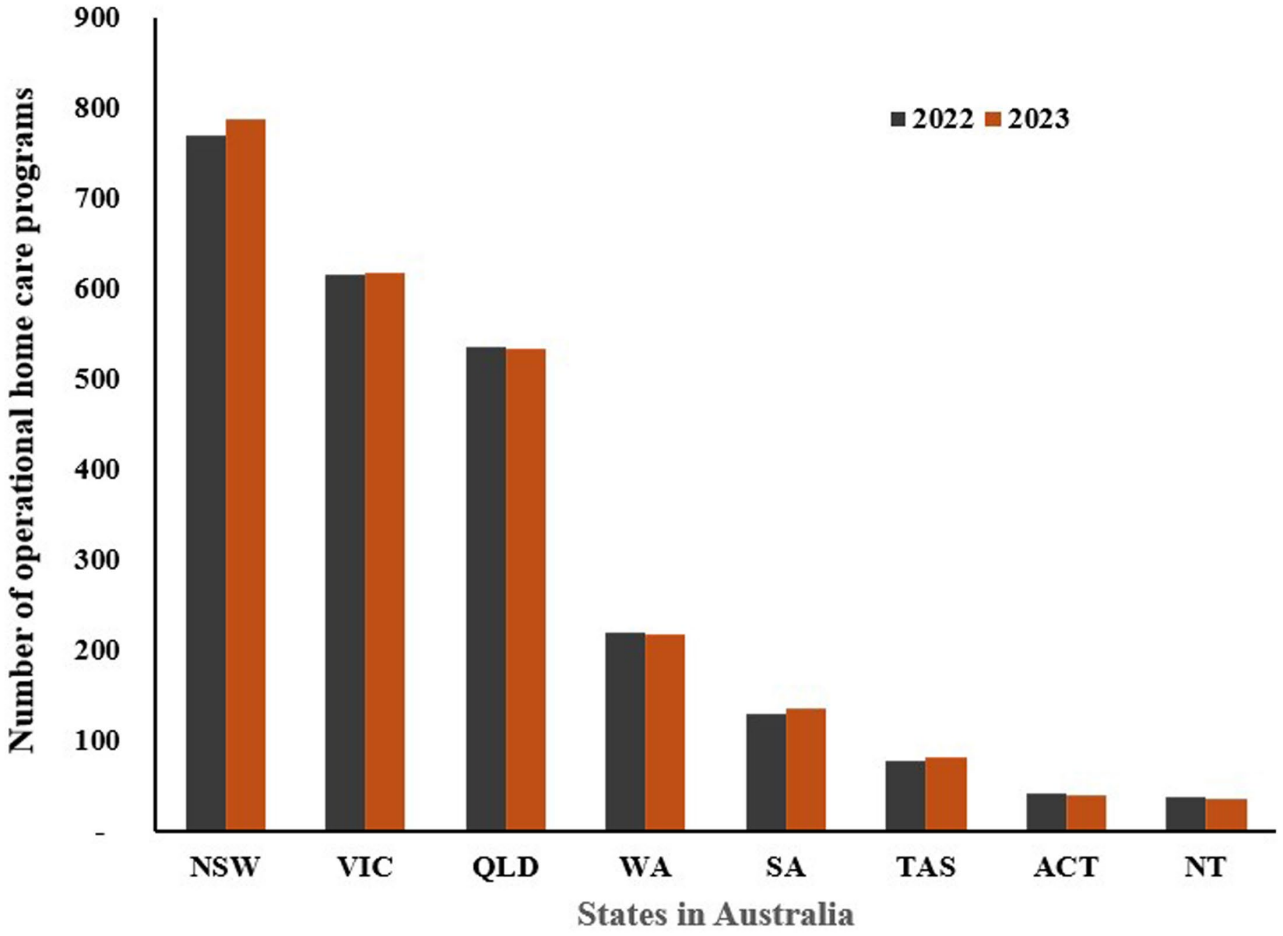
Number of home care programs in Australia, by state 2022–2023 (NSW: New South Wales, VIC: Victoria, QLD: Queensland, WA: Western Australia, SA: South Australia, TAS: Tasmania, ACT: Australian Capital Territory, NT: Northern Territory). Source: Department of Health and Aged Care (106, 107).

In contrast, the number of Home Care Programs generally increased, reflecting a growing trend towards in-home care services for the older adult (Figure 5) (106, 107). NSW saw an increase from 769 in 2022 to 788 in 2023, and VIC rose slightly from 615 to 618. SA and TAS also experienced increases in home care programs, from 130 to 135 and from 78 to 81, respectively. These trends suggest a shift towards providing more older adult care options that allow individuals to stay in their homes rather than move into residential care facilities. The preference for home-based care could be influenced by the desire for personalized care, comfort, flexibility, affordability, and safety (108). Overall, these numbers indicate a broader shift in Australia’s aged care landscape, with a gradual move towards expanding home care services while maintaining or slightly reducing the number of traditional residential care facilities. This trend reflects changing societal preferences, the aging population’s desire for independence, and potential policy reforms aimed at enhancing aged care quality and accessibility. The data underscores the need for adaptive strategies in aged care to meet the evolving needs and preferences of Australia’s older adult population.

#### 3.1.6 Workforce profile

The professionals delivering care work in government, for-profit, and not-for-profit organizations. Ris et al. emphasized that family caregivers are often overlooked but play a crucial role in providing care and complementing care by professionals (109). Family members help in decision-making on care, offer companionship, and monitor seniors’ health. Family caregivers serve as a bridge between the family of the aged and the care staff (110). Their input is critical to optimizing aged care in the sector. They also provide care beyond the scope of what health professionals can offer. Personal aged care workers assist older residents with daily living activities. They undertake household tasks, offer mobility support, provide meal support, and report any observed mood, behavior, and physical changes to healthcare professionals such as nurses (111). Their assistance significantly shapes the seniors’ well-being and quality of life. RNs assume a leading role in caregiving and ensuring seniors’ well-being (112). They deliver direct patient care, coordinate care delivery by other practitioners, and develop procedures to ensure regulatory compliance and quality care provision. RNs also offer patient advocacy and are involved in educating the public and family members about handling older residents (113). Apart from RNs, other health professionals such as physiotherapists and psychologists, contribute to aged care by maintaining seniors’ physical and mental well-being (114). The pursuit of holistic care also provides social workers and chaplains with opportunities to contribute to advancing aged care (115). The collaboration between providers, caregivers, and families is critical to effective age care delivery.

As the aged population increases, the rising demand for aged care services continues to change the workforce landscape in the sector. The Department of Health and Aged Care reported that the number of aged care workers in Australia is around 370,000 (116). Personal care workers form the largest (78%) portion of direct care workers in all aged care types (117). The 2020 Aged Care Workforce Census revealed that direct care staff mostly serve in permanent part-time positions. Around half of the direct care workers were aged below 40 years.

**Figure 6:**
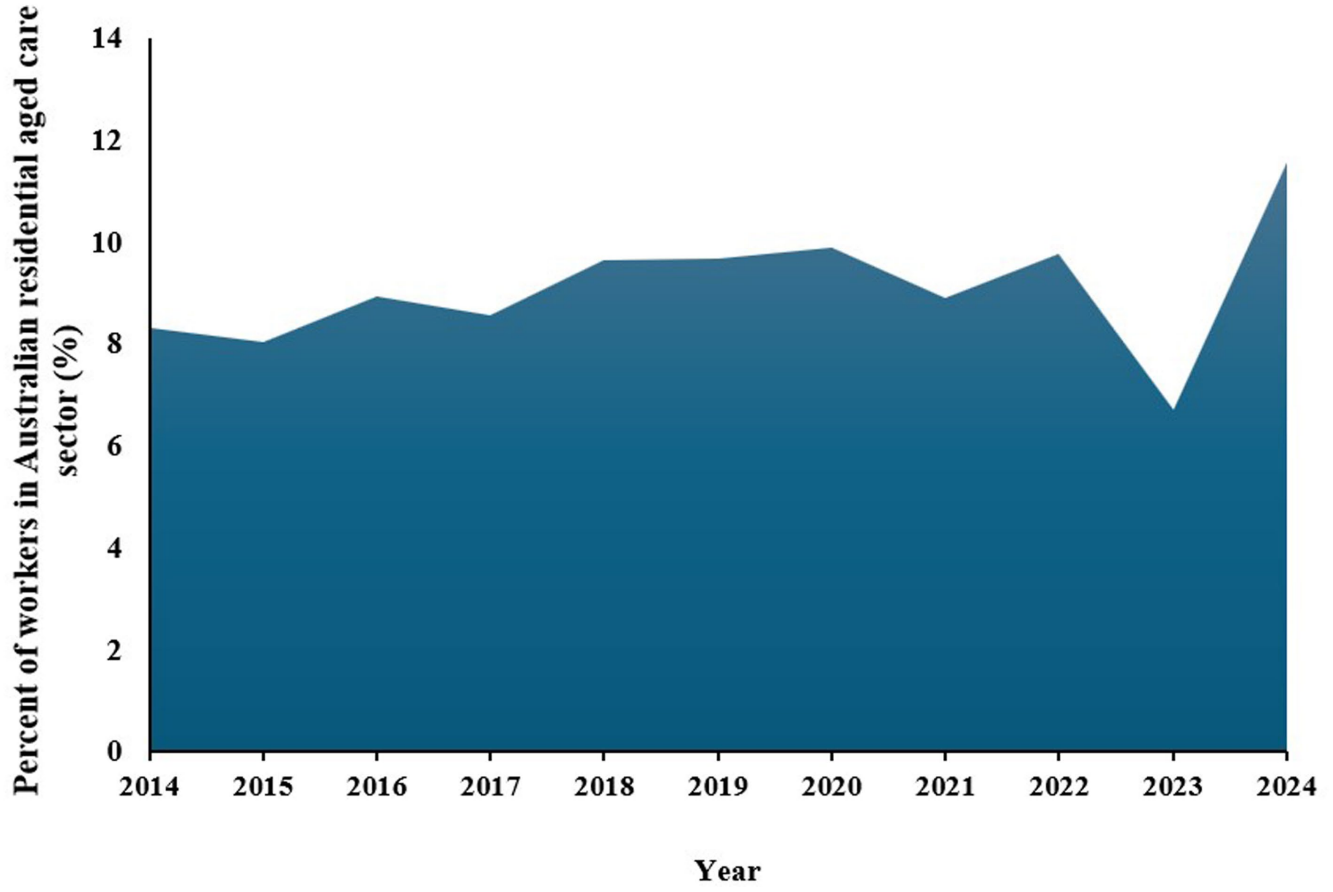
Percentage of employees in Australian aged care for the last 10 years. Source: Australian Institute of Health and Welfare (109).

Figure 6 provides insights into the percentage share of Australia’s total workforce employed in the residential aged care sector over the past decade, illustrating significant trends and fluctuations. From 2014 to 2024, there has been a general increase in the percentage of the workforce involved in aged care, starting at 8.31% in 2014 and rising to a peak of 11.57% in 2024 (118). This upward trend suggests that aged care has been growing as a key employment sector, likely driven by an aging population and increased demand for elder care services (119), highlighting the expanding importance of aged care in the Australian economy and reflecting the societal need to support an aging demographic. However, the data reveals a significant drop in 2023, where the percentage share fell sharply to 6.70%, marking the lowest point in the decade, caused by the impact of the COVID-19 pandemic (118, 119).

CHSP and Home Care Packages Program (HCPP) staff were on average older than those working in residential aged care (RAC). RNs comprise 76% of all nurses and physiotherapists accounted for 22% of allied health workers. A notable (55%) portion of direct care workers are employed permanently. Six percent served as subcontractors. However, the proportion of those on a subcontractor basis depended on the job role (117). Caution is necessary when considering the size of the workforce because the staff can serve across service care types or multiple providers.

Females dominate the sector. More direct care workers identify as female, but more allied health professionals identified as male than in other aged care roles. The proportion of male nurses in HCCP was 6 % and increased to 11% in the case of personal care workers. These were lower than the 14% representation in RAC for both nurses and PCWs. Moreover, the aged care sector staff have a diverse skill set. The 2020 census survey found that 63% of PCWs during the census had a Certificate III or higher in a direct care field. Other 4 % were studying for the same (117). Among HCPP providers, care managers are more likely to have a nursing background. During the 2020 survey, 352 providers employed nurses with bachelor’s degrees and 204 reported having care managers holding post-graduate nursing qualifications. Another 355 providers had care managers with qualifications in other areas that were not specified such as Certificate III in Individual Support (Ageing) and Community Service qualifications (117). These insights reveal how the roles are perceived in the labor market.

#### 3.1.7 Funding arrangements

##### 3.1.7.1 Finance generation in the Australian aged care industry

The funding mechanism for aged care reflects the intricacies that characterize federal-state financial relationships in Australia. The Commonwealth has greater tax-raising powers (120, 121). Therefore, it provides most of the funding for aged care services, even those operated by government, not-for-profit, and private organizations (122). The interplay of public and private interests is a feature. The federal government subsidizes services offering aged care and support, and private care services cover facility fees, accommodation, and other costs (60). The federal government determines the minimum prices that private insurers should pay. Medical fees are usually billed directly by providers under private practice arrangements (50). The funding by the federal government is set out in the Aged Care Act of 1997, which is responsible for providing legislative authority for care funding and price setting (84). The subsidies target consumers or providers for home care, flexible care, and long-term residential care, respectively.

The funding for aged care is based on care assessment in line with the Aged Care Financing Instrument (ACFI). The ACFI is a regulatory funding instrument that helps determine the profile of relative care needs profile and the required funding (123). An independent audit applies to the provided assessment. Residents pay care fees, which are then balanced with government subsidies. The paid amount depends on the assessment of a resident’s income and assets and the cost of care (124, 125). However, the government covers the full cost of care for low-income people with no assets. A sliding scale applies in determining what one pays. Fees that residents pay are regularly reviewed, and annual and lifetime caps exist to minimize the fees that residents have to pay (126). Residents determine whether to make a lump sum, a fully refundable payment, or a daily accommodation payment. A regulatory formula exists to help determine the daily payment to ensure the provider gets a return equivalent to the refundable lump sum (127, 128).

##### 3.1.7.2 Allocation of finances: providers and consumers

The aged care sector in Australia comprises diverse stakeholders. The Department of Health establishes fee and charge schedules by the Aged Care Act of 1997 and the Minister for Aged Care engages commissions to decide on matters concerning consumer information, provider accountability, fees and payments, subsidies, quality of care, and sanctions (129). There is a broad range of providers because the services offered vary (37). Government-subsidized aged care homes are run by approved providers. The Aged Care Act 1997 mandates the Department of Health to approve providers’ applications to deliver residential aged care (130). In this view, the providers may be in the form of for-profit providers, not-for-profit organizations, and government providers (131). The providers deliver different types of aged care. Apart from providers, the other critical segment of the sector is the consumers meeting eligibility criteria. Their needs vary, with some able to remain at home, others requiring full-time residential care, while others’ needs are flexible (132). Other pertinent stakeholders in the sector include the Independent Health and Aged Care Pricing Authority and the Aged Care Financing Authority (ACFA). The former is responsible for considering and approving accommodation payments and extra service fees exceeding the maximum payments set (133). ACFA provides the government with independent advice on funding. The committee conducts consultations with consumers and finance and aged care sectors before publishing an annual findings report that highlights developments, challenges, and issues facing the sector (64). There are various reports covering relevant statistics on aged care provision.

##### 3.1.7.3 Technology adoption in the provision of services

Technology has been having an increasingly significant role in the delivery of aged care in the Australian aged care sector. As a result, the aged care sector is undergoing a major shift as technology promises improvements in efficiency in care delivery (134). E-health offers substantial potential to enhance access to efficient and effective care for older adults (135). Technology eases care planning and medication (136), facilitates tracking of vital signs to inform intervention (137), and eases access to providers (138). The adoption of the Internet of Things (IoT) by older adults has been rising because they are perceived as essential and positive to their everyday lives.

As many people seek to live independently at home into advanced old age, technology provides an opportunity to enrich the lives of aged care residents. The aged care sector has increasingly embraced technologies promising to empower seniors’ well-being and independence (139). For instance, robotic pets are growing in popularity (140). Touch-screen tablet devices are gaining popularity because they support communication between seniors and their family members (141). Interest in virtual reality has also risen as providers seek to engage seniors in diversional activities that help foster reminiscence, enable them to experience places and activities they no longer experience, and foster calm and meditation to address anxiety and agitation (5). The potential benefits of technology are increasingly clear.

In addition, the increasing integration of technology facilitates data-driven care. Australia’s aged care system lags other OECD countries in quality assessment and reporting (142). Ludlow et al. criticized the limited and disparate quality information lowering the capacity to make informed care decisions (143). Nevertheless, there is a growing awareness of the need to leverage electronic systems to support evidence-based practice across aged care services (144, 145). Technologies help providers monitor and benchmark key quality indicators.

## 4 Discussion

### 4.1 Challenges of the Australian aged care industry

The Australian aged care sector is facing growing challenges including workforce crises, population ageing, and financial sustainability to support the sector. Additionally, the service sector is becoming highly complex and persistently struggles to deliver high-quality care to the rising number of older Australians. This paper identifies the key challenges based on the content and secondary data analysis.

#### 4.1.1 Aging burden

Improvements in healthcare and the continued aging of the large cohort of baby boomers are expected to maintain the growth in the number of older Australians. The key driver for increases in Australian care spending is the increased number of Australians aged above 80 (134). In their report, KPMG noted a growth in government expenditure correlating with the 24% rise year on year for those receiving home care services. There were 906 providers in June 2021, up from the 487 providers recorded in 2016 (146). The Intergenerational Report (2023) projected that aged adults will triple over the next four decades to over 3.5 million people. As the demand for the aged care system increases, a concerning trend is the declining availability of the workforce. The Committee for Economic Development of Australia projected that the aged care workforce will decline by over 110,000 workers by 2030 (147). The increase will exert considerable pressure on aged care spending.

#### 4.1.2 Financial sustainability

As the number of Australians expected to need aged care services increases, existing funding mechanisms are no longer sustainable with deteriorating health and high acuity. The capital needs in the next decade are projected to exceed $55 billion (148). The future supply of aged case accommodation will be constrained as the population continues to age. As the issue of financial sustainability continues, aged care providers are likely to slow down projects or place growth strategies on hold (149). However, Blackberry and Morris noted that concerns over capital needs may subside as older Australians increasingly choose home care packages and the number of seniors in residential care decreases (7). Identifying successful funding applications will be necessary to support infrastructure developments and increase program effectiveness (150). The government would need to focus funding on having a suitably sized and skilled workforce critical to the effectiveness and sustainability of any reforms (151). Rawlings et al. observed that the solution lies in streamlining the current diverse funding models and sources to effectively respond to the increasing demand facing the current imperfect and fragmented care system (152). Sustained funding is critical to maintaining care quality and minimizing negative care experiences.

#### 4.1.3 Workforce challenges

Care providers have a critical role in determining the effectiveness of the aged care system. The essence of their input is notable, as Peters et al. highlighted the need for minimum time standards and recommended an average of at least 258 min of care by mid-2026 in nursing homes (151). The delivery of aged care has been progressively evolving towards consumer-directed and person-centered care, which prioritizes the autonomy and individual preferences of residents. To sustain this momentum of reinventing aged care, ongoing efforts should focus on promoting holistic and fulfilling lives for older adults within these settings. A critical component in achieving truly innovative design practices lies in harnessing the potential of advanced technologies. Therefore, it is imperative for aged care staff to cultivate the necessary skills to facilitate the integration of emerging technological innovations that can enable consumers’ independence, support health and safety, and enhance mobility (153). Rostgaard et al. have advocated for reablement strategies that call for the adoption of novel methods in aged service delivery. Reablement promotes the development of innovative roles, more effective fulfilment of individual needs, enhanced collaborations, and evolving responsibilities among individuals, their families, and care practitioners (154). It is crucial that innovative aged care solutions be both cost-effective and highly individualized to ensure optimal outcomes for all stakeholders involved in the care process.

#### 4.1.4 Adaption to emerging technologies

The integration of emerging technologies presents unique opportunities to revolutionize aged care. Digital innovations have demonstrated the potential to improve the social, cognitive, and emotional well-being of older adults (155). As the aging population continues to strain existing aged care systems, technological advancements, such as wearable devices and the IoT, can alleviate pressure by enabling more efficient healthcare services (156). The strategic combination of social robotics, smart devices, IoT, and surveillance systems holds the potential to enhance independence and decrease reliance on aged care facilities.

Although robots cannot manage residential homes, reconsidering the design and delivery of care is crucial given the demographic shift (153). To maximize the benefits of digital technologies, identifying factors that encourage seniors’ adoption is essential (155). Effective integration of technology will pave the way for developing technology contextually appropriate solutions that address the escalating shortages in the aged care workforce.

## 5 Conclusion

Australia’s aging population places a significant strain on the aged care sector, with a growing number of individuals aged 65 and older. The Australian government has introduced various reforms to address the needs and rising expectations of older citizens. Nonetheless, challenges persist, such as workforce shortages, unsustainable financing mechanisms, the aging burden, and adapting to emerging technologies, impacting the quality of care provided to older Australians. This review paper provides a comprehensive overview of Australia’s aged care system, highlighting the high proportion of older adults receiving care. Discontent with aged care homes has led to a preference for in-home care among older adults receiving care. The growing aging population necessitates governments to ensure sustainable long-term care delivery, promoting healthy aging and enhancing the quality of life for older adults. While Australia invests heavily in aged care, issues like abuse, neglect, quality, and standard care delivery persist amid funding, governance, and accountability concerns.

Addressing these challenges necessitates focusing on quality concerns, dysfunction, neglect, and abuse alongside the legal and regulatory environment. Achieving this requires reforms targeting funding, governance, organizational structures, and internal processes as the older adult population grows. Such reforms would pave the way for a more effective, accountable, and financially sustainable aged care sector in Australia.

## Data Availability

The original contributions presented in the study are included in the article/supplementary material, further inquiries can be directed to the corresponding author.
